# Investigations into the construction of the penta­substituted ring *C* of Neosurugatoxin – a crystallographic study

**DOI:** 10.1107/S2056989015023506

**Published:** 2016-01-01

**Authors:** Alan M. Jones, John M. D. Storey, William T A Harrison

**Affiliations:** a Department of Chemistry, University of Aberdeen, Meston Walk, Aberdeen AB24 3UE, Scotland; b Division of Chemistry and Environmental Science, School of Science and the Environment, Faculty of Science and Engineering, Manchester Metropolitan University, John Dalton Building, Chester Street, Manchester M1 5GD, England

**Keywords:** Fused rings, conformation, intra­molecular O—H⋯O hydrogen bonds, weak inter­actions, crystal structure

## Abstract

The crystal structures of three cyclo­penta­[*c*]furans with various substituents at the 4-, 5- and 6-positions of the ring system are reported, namely, (±)-(3a*R*,4*S*,5*S*,6a*S*)-4-methyl-5-phenyl­hexa­hydro-1*H*-cyclo­penta­[*c*]furan-4,5-diol, C_14_H_18_O_3_, (I), (±)-(3a*R*,4*S*,5*S*,6a*S*)-4-benz­yloxy-4-methyl-5-phenyl­hexa­hydro-1*H*-cyclo­penta­[*c*]furan-5-ol, C_21_H_24_O_3_, (II), and (±)-(1a*R*,1b*S*,4a*R*,5*S*,5a*R*)-5-benz­yloxy-5-methyl-5a-phenyl­hexa­hydro-2*H*-oxireno[2′,3′:3,4]cyclopenta­[1,2-*c*]furan, C_21_H_22_O_3_, (III). The dominant inter­action in (I) and (II) is an O—H⋯O hydrogen bond across the bicyclic 5,5-ring system between the non-functionalized hy­droxy group and the tetra­hydro­furan O atom, which appears to influence the envelope conformations of the fused five-membered rings, whereas in (III), the rings have different conformations. A weak intra­molecular C—H⋯O inter­action appears to influence the degree of tilt of the phenyl ring attached to the 5-position and is different in (I) compared to (II) and (III).

## Abstract 2

The mol­ecular conformations of three highly substituted cyclo­penta­[*c*]furans appear to correlate strongly with different intra­molecular O—H⋯O and C—H⋯O inter­actions.

## Chemical context   

Neosurugatoxin, C_30_H_34_BrN_5_O_15_, is the causative agent behind the toxicity of the Japanese ivory shell, *Babylonia Japonica*, a shellfish widely consumed in Japan. Neosurugatoxin, shown in Scheme 1 below, was first isolated and the structure delineated using X-ray crystallographic studies by Kosuge and co-workers (Kosuge *et al.*, 1981 ▸, 1982 ▸). 
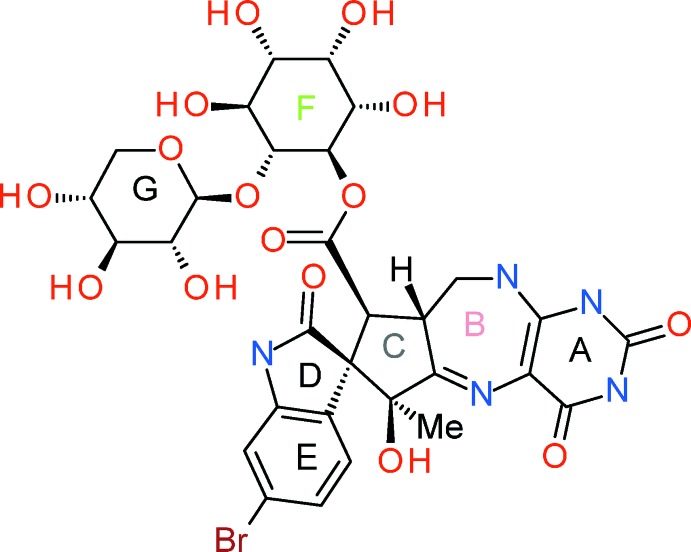


Biological studies with Neosurugatoxin have shown it to have a wide range of actions on the central nervous system including: potent nicotinic acetyl­choline receptor antagonist (Yamada *et al.*, 1988 ▸; Bai & Sattelle, 1993 ▸; Tornøe *et al.*, 1995 ▸); inhibition of acetyl­choline release and blockage of muscle and neuronal nicotinic receptors (Hong *et al.*, 1992 ▸); and a central action upon nicotinic cholinoreceptors (Bisset *et al.*, 1992 ▸). Alternative total syntheses of Neosurugatoxin have previously been reported by the Inoue and Okada groups (Inoue *et al.*, 1986 ▸, 1994 ▸; Okada *et al.*, 1989 ▸). Intrigued by the dense functionality and complexity of ring *C* in Neosurugatoxin (see Scheme 1), we investigated a synthetic route to novel simplified cyclo­penta­nes with diversity vectors to install the required functionality at a later stage.
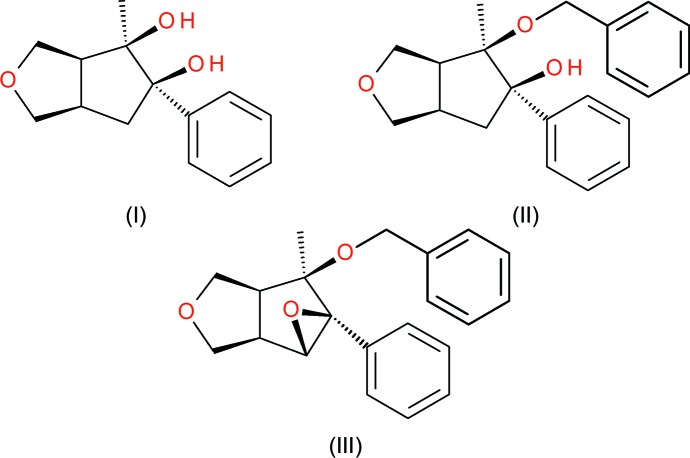


As part of these studies, we now report the crystal structures of three of these compounds, namely (±)-(3a*R*,4*S*,5*S*,6a*S*)-4-methyl-5-phenyl­hexa­hydro-1*H*-cyclo­penta­[*c*]furan-4,5-diol, C_14_H_18_O_3_, (I), (±)-(3a*R*,4*S*,5*S*,6a*S*)-4-benz­yloxy-4-methyl-5-phenyl­hexa­hydro-1*H*-cyclo­penta­[*c*]furan-5-ol, C_21_H_24_O_3_, (II), and (±)-(1a*R*,1b*S*,4a*R*,5*S*,5a*R*)-5-benz­yloxy-5-methyl-5a-phenyl­hexa­hydro-2*H*-oxireno[2′,3′:3,4]cyclo­penta­[1,2-*c*]furan, C_21_H_22_O_3_, (III), see Scheme 2 above.

## Structural commentary   

**Figure 1:**
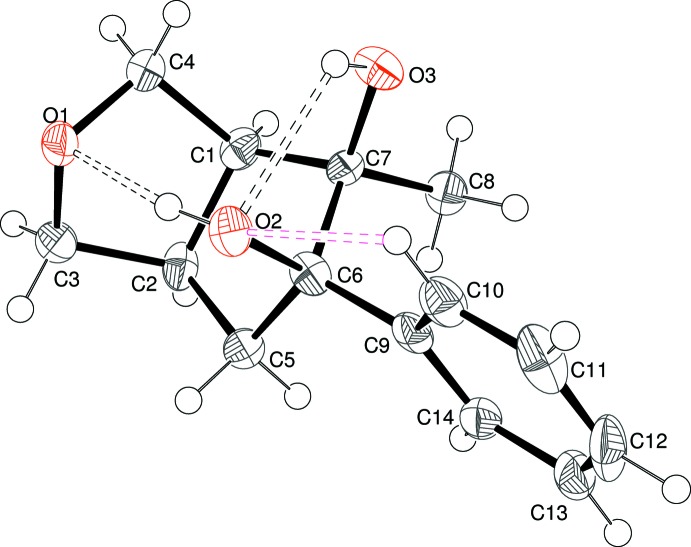
The mol­ecular structure of (I), showing 50% probability displacement ellipsoids. Intra­molecular O—H⋯O and C—H⋯O inter­actions are shown as black and pink double-dashed lines, respectively.

Compound (I) crystallizes in the centrosymmetric space group *Pbca* and its mol­ecular structure is illustrated in Fig. 1 ▸. In the arbitrarily chosen asymmetric mol­ecule, the configurations of the stereogenic atoms C1, C2, C6 and C7 are *S*, *R*, *R*, and *R*, respectively. As expected, the junction of the fused rings is *cis* (H1—C1—C2—H2 = 5°). The C1/C2/C3/O1/C4 ring has an envelope conformation, with O1 displaced from the mean plane of the carbon atoms (r.m.s. deviation = 0.018 Å) by 0.566 (5) Å. The C1/C2/C5/C6/C7 ring also has an envelope conformation, with C6 displaced from the other atoms (r.m.s. deviation = 0.026 Å) by 0.573 (6) Å. The dihedral angle between the almost planar parts of the rings is 58.3 (2)°: the overall shape could be described as a butterfly, with the flap atoms (O1 and C6) pointing inwards. Atoms O2 and O3 lie to the same face of the ring although there is a significant twist between them [O2—C6—C7—O3 = 46.5 (4)°]. The O2—C6—C7—C8 torsion angle is 164.9 (3)° and the C8—C7—C6—C9 torsion angle is 47.6 (4)°. The dihedral angle between the pendant benzene ring (C9–C14) and C1/C2/C5/C7 is 64.00 (17)°. The mol­ecular structure of (I) features two intra­molecular O—H⋯O hydrogen bonds (Table 1 ▸). The O3—H3*o*⋯O2 bond closes an *S*(5) ring. The O2—H2*o*⋯O1 bond, which bridges across the top of the fused-ring system to generate an *S*(7) ring, may influence the conformations of the five-membered rings. An intra­molecular C10—H10⋯O2 short contact (H⋯O = 2.33 Å) is also present: although the C—H⋯O angle of 100° is extremely small to be regarded as a bond (Steiner, 1996 ▸) it is inter­esting to compare this C—H grouping to the situation in (II) and (III) (*vide infra*).

**Figure 2:**
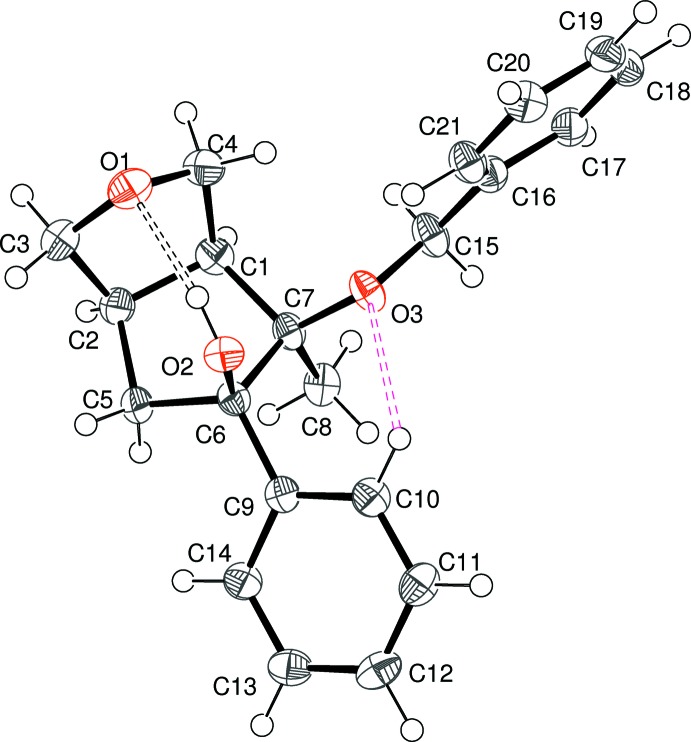
The mol­ecular structure of (II), showing 50% probability displacement ellipsoids. Intra­molecular O—H⋯O and C—H⋯O inter­actions are shown as black and pink double-dashed lines, respectively.

The asymmetric unit of (II), which crystallizes in the centrosymmetric space group *P*2_1_/*c*, contains one mol­ecule (Fig. 2 ▸): for ease of comparison with (I), the stereogenic centres in this mol­ecule have configurations of *S*, *R*, *R*, and *R*, for C1, C2, C7 and C8, respectively. As with (I), the C1/C2/C3/O1/C4 ring has an envelope conformation, with O1 as the flap, displaced by 0.571 (2) Å from the other atoms. The conformation of the C1/C2/C5/C6/C7 ring in (II) is also an envelope, with C6 as the flap [displacement = 0.618 (2) Å]. The dihedral angle between C1/C2/C3/C4 (r.m.s. deviation = 0.004 Å) and C1/C2/C5/C7 (r.m.s. deviation = 0.016 Å) of 58.28 (7)° is identical to the equivalent value for (I) and the flap atoms (O1 and C6) also point inwards. Key torsion angles in (II) include O2—C6—C7—O3 [42.19 (17)°], O2—C6—C7—C8 [164.41 (13)°] and C8—C7—C6—C9 [46.42 (17)°]: these data are similar to the corresponding values for (I). However, the dihedral angle between the C9–C14 benzene ring and C1/C2/C5/C7 in (II) is 34.90 (9)°, which differs by some 30° compared to the equivalent value for (I). The dihedral angle between the aromatic rings (C9–C14 and C16–C21) is 89.74 (5)°. As with (I), the hy­droxy (O2—H2*o*) grouping forms an intra­molecular hydrogen bond (Table 2 ▸) to O1 across the fused-ring system and an *S*(7) ring results. The C10—H10 grouping in (II) points towards O3 rather than O2 (H⋯O = 2.56 Å), which appears to correlate with the different orientation of the C9–C14 ring.

**Figure 3:**
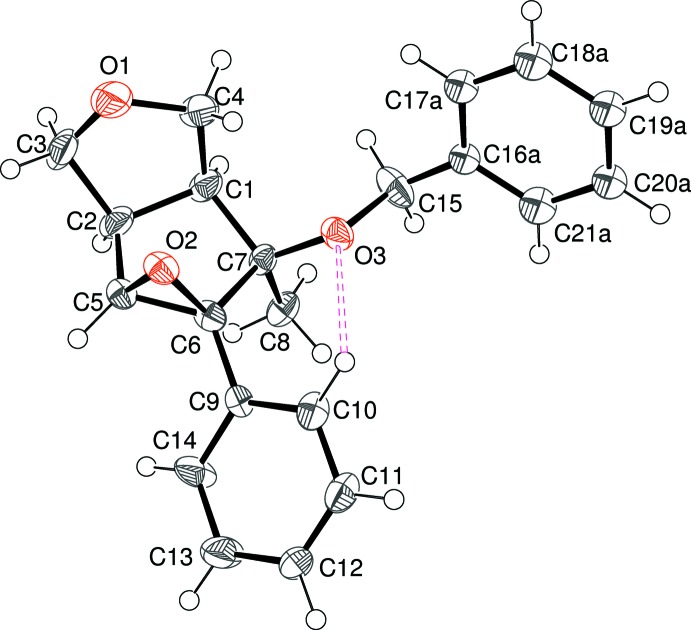
The mol­ecular structure of (III), showing 50% probability displacement ellipsoids. Only one orientation of the disordered C16–C21 benzene ring is shown. The intra­molecular C—H⋯O inter­action is shown as a pink double-dashed line.

Compound (III) crystallizes in the chiral space group *P*2_1_2_1_2_1_. The absolute structure was indeterminate in the present experiment and C1, C2, C5, C6 and C7 in the asymmetric mol­ecule were assigned configurations of *S*, *R*, *S*, *S* and *R*, respectively (Fig. 3 ▸). Based on the synthesis, we assume the bulk sample to be racemic. The conformation of the C1/C2/C3/O1/C4 ring is different to the equivalent unit in (I) and (II): in (III), this ring is twisted about the C2—C3 bond [*Q*(2) = 0.307 (10) Å, φ(2) = 232.5 (18)°] such that C2 and C3 are displaced from the O1/C4/C1 plane by −0.22 (2) and 0.29 (2) Å, respectively. The C1/C2/C5/C6/C7 conformation in (III) is an envelope, but the flap atom is different to that in (I) and (II): in this case C1 (rather than C6) is displaced by 0.487 (14) Å from the other atoms (r.m.s. deviation = 0.011 Å). The dihedral angle between the five-membered rings (all atoms) of 69.6 (5)° in (III) is significantly larger than the corresponding angle for (I) and (II). The epoxide ring (C5/C6/O2) subtends a dihedral angle of 74.0 (4)° with respect to C2/C5/C6/C7. Important torsion angles in (III) include O2—C6—C7—O3 [76.3 (8)°], O2—C6—C7—C8 [–161.3 (6)°] and C8—C7—C6—C9 [55.4 (9)°]: these data are very different from the corresponding values for (I) and (II), which must in part be due to the steric inflexibility of the epoxide ring containing O2. The dihedral angle between the C9–C14 benzene ring and C2/C5/C6/C7 in (II) is 49.3 (4)°, which is inter­mediate between the corresponding values for (I) and (II). The dihedral angle between the C9–C14 and C16a–C21a benzene rings is 41.0 (7)°. There are obviously no classical intra­molecular hydrogen bonds in (III), but, as in (II), a C10—H10⋯O3 link (Table 3 ▸) is seen.

## Supra­molecular features   

**Figure 4:**
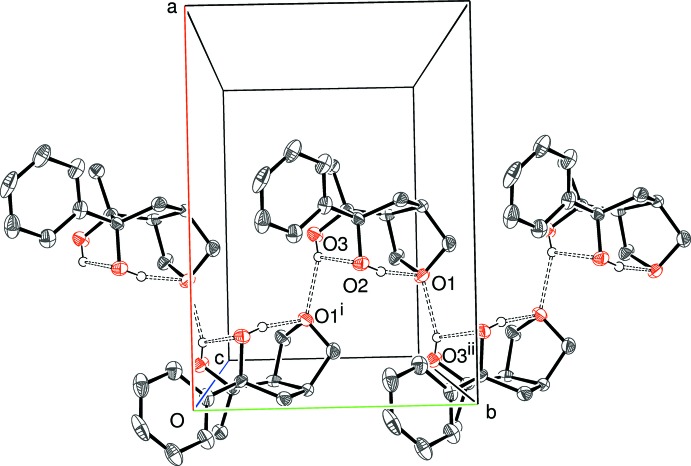
Partial packing diagram for (I), showing the formation of [100] chains linked by O—H⋯O hydrogen bonds (double-dashed lines). Symmetry codes as in Table 1 ▸. All C-bonded H atoms have been omitted for clarity.

**Figure 5:**
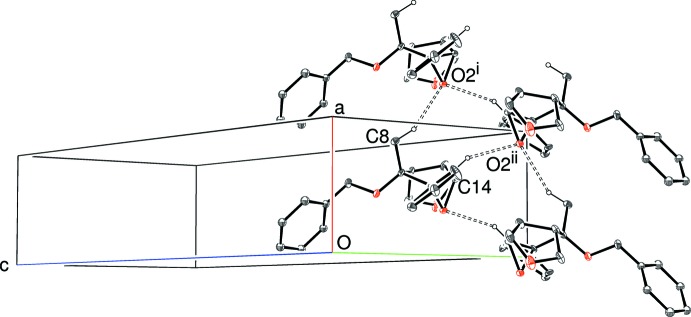
Partial packing diagram for (III), showing the formation of [100] chains linked by C—H⋯O hydrogen bonds (double-dashed lines). Symmetry codes as in Table 3 ▸. All H atoms except those involved in the C—H⋯O bonds have been omitted for clarity.

In the crystal of (I), the mol­ecules are linked into [010] chains by O3—H3*o*⋯O1^i^ [symmetry code: (i) 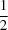 − *x*, *y* − 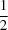, *z*] hydrogen bonds (Table 1 ▸, Fig. 4 ▸): the same OH group also participates in an intra­molecular bond, as described above. Adjacent mol­ecules are enanti­omers, being related by *b*-glide symmetry and the chain has a *C*(6) motif. Long and presumably very weak inter­molecular C—H⋯O and C—H⋯π inter­actions (Tables 2 ▸ and 3 ▸) are observed in the crystals of (II) and (III). Assuming these inter­actions to be significant, (100) sheets in (II) and [100] chains in (III) arise (Fig. 5 ▸). It is notable that the epoxide O atom accepts both C—H⋯O inter­actions in the latter. Aromatic π–π stacking is absent in these structures, the shortest centroid–centroid separations being *ca* 4.97 in (I), 5.03 in (II) and 5.24 Å in (III).

## Database survey   

A search of the Cambridge Structural Database (Groom & Allen, 2014 ▸) for compounds with a cyclo­penta­[*c*]furan skeleton revealed 321 matches; of these, just two had O atoms bonded to the 4- and 5-positions of the fused-ring system, *viz*.: VALFIX (Dumdei *et al.*, 1989 ▸) and YEYBEB (Wang *et al.*, 2012 ▸), but otherwise, neither bears a close resemblance to the compounds described here.

## Synthesis and crystallization   

Full synthesis details will be reported in due course, but a summary of the steps followed to prepare (I), (II) and (III) are detailed as follows. A Pauson–Khand [2 + 2 + 1] cyclo­addition (Pauson, 1985 ▸) was used to prepare the key starting material: a mixture of phenyl­acetyl­ene, 2,5-di­hydro­furan and dicobalt octa­carbonyl in toluene under an inert atmosphere was heated to reflux for 1 h to afford (±)-(3a*R*,6a*S*)-5-phenyl-1,3,3a,6a-tetra­hydro-4*H*-cyclo­penta­[*c*]furan-4-one, *A* [Chem scheme3]: after purification by silica gel chromatography, spectroscopic data were in accordance with those previously reported by Brown *et al.* (2005 ▸). Treatment of *A* [Chem scheme3] with methyl magnesium iodide in anhydrous tetra­hydro­furan using the procedure of Coote *et al.* (2008 ▸) afforded (±)-(3a*R*,4*S*,6a*S*)-4-methyl-5-phenyl-3,3a,4,6a-tetra­hydro-1*H*-cyclo­penta­[*c*]furan-4-ol, *B* [Chem scheme3]. Treatment of *B* [Chem scheme3] with *m*-CPBA in anhydrous di­chloro­methane at 273 K yielded (±)-(1a*R*,1b*S*,4a*R*,5*S*,5a*R*)-5-methyl-5a-phenyl­hexa­hydro-2*H*-oxireno[2′,3′:3,4]cyclo­penta­[1,2-*c*]furan-5-ol, *C* [Chem scheme3], with facial selectivity directed by the hy­droxy group (Langston *et al.*, 2007 ▸). Treatment of *C* with lithium aluminium hydride in anhydrous tetra­hydro­furan (Howe *et al.*, 1987 ▸) afforded the epoxide opened product, (±)-(3a*R*,4*S*,5*S*,6a*S*)-4-methyl-5-phenyl­hexa­hydro-1*H*-cyclo­penta­[*c*]furan-4,5-diol, (I). Further treatment of (I) with benzyl chloride under identical conditions to above afforded (±)-(3a*R*,4*S*,5*S*,6a*S*)-4-(benz­yloxy)-4-methyl-5-phenyl­hexa­hydro-1*H*-cyclo­penta­[*c*]furan-5-ol, (II). Benzyl­ation of *C* using the procedure of Peng & Woerpel (2003 ▸) afforded (±)-(1a*R*,1b*S*,4a*R*,5*S*,5a*R*)-5-(benz­yloxy)-5-methyl-5a-phenyl­hexa­hydro-2*H*-oxireno[2′,3′:3,4]cyclo­penta­[1,2-*c*]furan, (III).
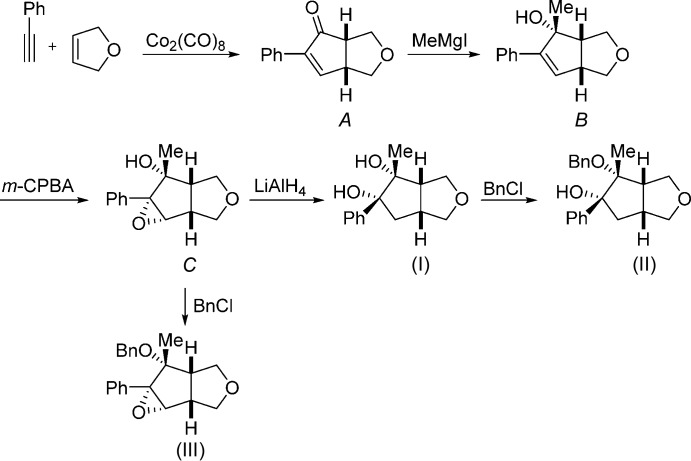


## Refinement   

Crystal data, data collection and structure refinement details for (I)–(III) are summarized in Table 4 ▸. The O-bound H atoms were located in difference maps and their positions freely refined. The C-bound H atoms were geometrically placed (C—H = 0.95–1.00 Å) and refined as riding atoms. The constraint *U*_iso_(H) = 1.2*U*_eq_(carrier) or 1.5*U*_eq_(methyl carrier) was applied in all cases. The methyl H atoms were allowed to rotate, but not to tip, to best fit the electron density. The C16–C21 benzene ring in (III) was modelled as being disordered over two overlapped orientations in a 0.54 (3):0.46 (3) ratio; the rings were constrained to be regular hexa­gons (C—C = 1.39 Å). The crystal quality for (I) and (III) was poor, which may correlate with the rather high *R*-factors obtained, although the structures are clearly resolved with acceptable geometrical precision. The absolute structure of compound (III) was indeterminate in the present experiment.

## Supplementary Material

Crystal structure: contains datablock(s) I, II, III, global. DOI: 10.1107/S2056989015023506/gk2648sup1.cif

Structure factors: contains datablock(s) I. DOI: 10.1107/S2056989015023506/gk2648Isup2.hkl

Structure factors: contains datablock(s) II. DOI: 10.1107/S2056989015023506/gk2648IIsup3.hkl

Structure factors: contains datablock(s) III. DOI: 10.1107/S2056989015023506/gk2648IIIsup4.hkl

Supporting information file. DOI: 10.1107/S2056989015023506/gk2648Isup5.cml

Supporting information file. DOI: 10.1107/S2056989015023506/gk2648IIsup6.cml

Supporting information file. DOI: 10.1107/S2056989015023506/gk2648IIIsup7.cml

CCDC references: 1440873, 1440872, 1440871

Additional supporting information:  crystallographic information; 3D view; checkCIF report

## Figures and Tables

**Table 1:** Hydrogen-bond geometry (Å, °) for (I)

*D*—H⋯*A*	*D*—H	H⋯*A*	*D*⋯*A*	*D*—H⋯*A*
O2—H2*o*⋯O1	0.84 (4)	1.96 (4)	2.776 (4)	163 (4)
O3—H3*o*⋯O1^i^	0.80 (4)	2.11 (4)	2.844 (4)	151 (4)
O3—H3*o*⋯O2	0.80 (4)	2.28 (4)	2.744 (3)	118 (4)
C10—H10⋯O2	0.95	2.33	2.667 (5)	100

Symmetry code: (i) 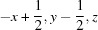.

**Table 2:** Hydrogen-bond geometry (Å, °) for (II) *Cg*4 is the centroid of the C16–C21 ring.

*D*—H⋯*A*	*D*—H	H⋯*A*	*D*⋯*A*	*D*—H⋯*A*
O2—H2*o*⋯O1	0.87 (2)	1.93 (2)	2.7794 (17)	162.9 (18)
C10—H10⋯O3	0.95	2.56	3.091 (2)	116
C5—H5*A*⋯O2^i^	0.99	2.58	3.266 (2)	126
C19—H19⋯O1^ii^	0.95	2.58	3.344 (2)	138
C12—H12⋯*Cg*4^iii^	0.95	2.74	3.6619 (19)	165

Symmetry codes: (i) 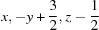; (ii) 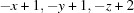; (iii) 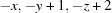.

**Table 3:** Hydrogen-bond geometry (Å, °) for (III) *Cg*6 is the centroid of the C16a–C21a ring.

*D*—H⋯*A*	*D*—H	H⋯*A*	*D*⋯*A*	*D*—H⋯*A*
C10—H10⋯O3	0.95	2.57	3.124 (10)	117
C8—H8*B*⋯O2^i^	0.98	2.58	3.462 (10)	150
C14—H14⋯O2^ii^	0.95	2.57	3.450 (11)	155
C4—H4*B*⋯*Cg*6^iii^	0.99	2.65	3.569 (10)	154

Symmetry codes: (i) 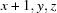; (ii) 
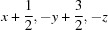
; (iii) 
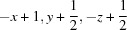
.

**Table 4:** Experimental details

	(I)	(II)	(III)
Crystal data			
Chemical formula	C_14_H_18_O_3_	C_21_H_24_O_3_	C_21_H_22_O_3_
*M*_r_	234.28	324.40	322.39
Crystal system, space group	Orthorhombic, *P* *b* *c* *a*	Monoclinic, *P*2_1_/*c*	Orthorhombic, *P*2_1_2_1_2_1_
Temperature (K)	120	120	120
*a*, *b*, *c* (Å)	10.997 (2), 7.7489 (9), 27.852 (4)	12.8872 (3), 19.3544 (6), 6.8046 (1)	5.6392 (2), 11.0427 (5), 26.6311 (13)
α, β, γ (°)	90, 90, 90	90, 92.3907 (16), 90	90, 90, 90
*V* (Å^3^)	2373.4 (6)	1695.75 (7)	1658.37 (13)
*Z*	8	4	4
Radiation type	Mo *K*α	Mo *K*α	Mo *K*α
μ (mm^−1^)	0.09	0.08	0.09
Crystal size (mm)	0.18 × 0.08 × 0.02	0.14 × 0.10 × 0.04	0.34 × 0.14 × 0.04

Data collection			
Diffractometer	Nonius KappaCCD	Nonius KappaCCD	Nonius KappaCCD
No. of measured, independent and observed [*I* > 2σ(*I*)] reflections	13446, 2312, 1303	28187, 3899, 2834	12562, 2221, 1867
*R*_int_	0.137	0.091	0.073
(sin θ/λ)_max_ (Å^−1^)	0.617	0.651	0.650

Refinement			
*R*[*F* ^2^ > 2σ(*F* ^2^)], *wR*(*F* ^2^), *S*	0.095, 0.148, 1.09	0.053, 0.132, 1.06	0.123, 0.279, 1.17
No. of reflections	2312	3899	2221
No. of parameters	161	222	190
H-atom treatment	H atoms treated by a mixture of independent and constrained refinement	H atoms treated by a mixture of independent and constrained refinement	H-atom parameters constrained
			
Δρ_max_, Δρ_min_ (e Å^−3^)	0.25, −0.27	0.30, −0.23	0.40, −0.44

Computer programs: *COLLECT* (Nonius, 1998 ▸), *DENZO* and *SCALEPACK* (Otwinowski & Minor, 1997 ▸), and *SORTAV* (Blessing, 1995 ▸), *SHELXS97* and *SHELXL97* (Sheldrick, 2008 ▸) and *ORTEP-3 for Windows* (Farrugia, 2012 ▸).

## supplementary crystallographic information

### (I) (±)-(3aR,4S,5S,6aS)-4-Methyl-5-phenylhexahydro-1H-cyclopenta[c]furan-4,5-diol Crystal data

**Table:**

C_14_H_18_O_3_	*D*_x_ = 1.311 Mg m^−^^3^
*M**_r_* = 234.28	Mo *K*α radiation, λ = 0.71073 Å
Orthorhombic, *P**b**c**a*	Cell parameters from 4280 reflections
*a* = 10.997 (2) Å	θ = 2.9–27.5°
*b* = 7.7489 (9) Å	µ = 0.09 mm^−^^1^
*c* = 27.852 (4) Å	*T* = 120 K
*V* = 2373.4 (6) Å^3^	Lath, colourless
*Z* = 8	0.18 × 0.08 × 0.02 mm
*F*(000) = 1008	

### (I) (±)-(3aR,4S,5S,6aS)-4-Methyl-5-phenylhexahydro-1H-cyclopenta[c]furan-4,5-diol Data collection

**Table:**

Nonius KappaCCD diffractometer	1303 reflections with *I* > 2σ(*I*)
Radiation source: fine-focus sealed tube	*R*_int_ = 0.137
Graphite monochromator	θ_max_ = 26.0°, θ_min_ = 3.3°
ω scans	*h* = −13→13
13446 measured reflections	*k* = −6→9
2312 independent reflections	*l* = −34→34

### (I) (±)-(3aR,4S,5S,6aS)-4-Methyl-5-phenylhexahydro-1H-cyclopenta[c]furan-4,5-diol Refinement

**Table:**

Refinement on *F*^2^	Primary atom site location: structure-invariant direct methods
Least-squares matrix: full	Secondary atom site location: difference Fourier map
*R*[*F*^2^ > 2σ(*F*^2^)] = 0.095	Hydrogen site location: inferred from neighbouring sites
*wR*(*F*^2^) = 0.148	H atoms treated by a mixture of independent and constrained refinement
*S* = 1.09	*w* = 1/[σ^2^(*F*_o_^2^) + (0.0231*P*)^2^ + 2.9549*P*] where *P* = (*F*_o_^2^ + 2*F*_c_^2^)/3
2312 reflections	(Δ/σ)_max_ < 0.001
161 parameters	Δρ_max_ = 0.25 e Å^−^^3^
0 restraints	Δρ_min_ = −0.27 e Å^−^^3^

### (I) (±)-(3aR,4S,5S,6aS)-4-Methyl-5-phenylhexahydro-1H-cyclopenta[c]furan-4,5-diol Special details

**Table:**

Geometry. All esds (except the esd in the dihedral angle between two l.s. planes) are estimated using the full covariance matrix. The cell esds are taken into account individually in the estimation of esds in distances, angles and torsion angles; correlations between esds in cell parameters are only used when they are defined by crystal symmetry. An approximate (isotropic) treatment of cell esds is used for estimating esds involving l.s. planes.
Refinement. Refinement of F^2^ against ALL reflections. The weighted R-factor wR and goodness of fit S are based on F^2^, conventional R-factors R are based on F, with F set to zero for negative F^2^. The threshold expression of F^2^ > 2sigma(F^2^) is used only for calculating R-factors(gt) etc. and is not relevant to the choice of reflections for refinement. R-factors based on F^2^ are statistically about twice as large as those based on F, and R- factors based on ALL data will be even larger.

### (I) (±)-(3aR,4S,5S,6aS)-4-Methyl-5-phenylhexahydro-1H-cyclopenta[c]furan-4,5-diol Fractional atomic coordinates and isotropic or equivalent isotropic displacement parameters (Å^2^)

**Table:**

	*x*	*y*	*z*	*U*_iso_*/*U*_eq_	
C1	0.4979 (4)	0.7625 (5)	0.44924 (12)	0.0240 (10)	
H1	0.5579	0.7736	0.4760	0.029*	
C2	0.5209 (4)	0.9051 (4)	0.41050 (13)	0.0251 (10)	
H2	0.5951	0.9739	0.4185	0.030*	
C3	0.4068 (4)	1.0179 (5)	0.41288 (13)	0.0285 (10)	
H3A	0.4189	1.1156	0.4353	0.034*	
H3B	0.3863	1.0641	0.3808	0.034*	
C4	0.3697 (4)	0.8034 (5)	0.46709 (13)	0.0292 (11)	
H4A	0.3237	0.6955	0.4728	0.035*	
H4B	0.3733	0.8695	0.4975	0.035*	
C5	0.5380 (4)	0.8075 (4)	0.36269 (12)	0.0252 (10)	
H5A	0.4957	0.8687	0.3363	0.030*	
H5B	0.6254	0.7990	0.3546	0.030*	
C6	0.4840 (4)	0.6287 (4)	0.36962 (12)	0.0221 (9)	
C7	0.5147 (4)	0.5877 (4)	0.42306 (12)	0.0207 (9)	
C8	0.6451 (4)	0.5287 (5)	0.43001 (12)	0.0254 (10)	
H8A	0.6578	0.4196	0.4129	0.038*	
H8B	0.6610	0.5120	0.4643	0.038*	
H8C	0.7006	0.6164	0.4173	0.038*	
C9	0.5241 (4)	0.4920 (5)	0.33435 (11)	0.0235 (10)	
C10	0.4430 (4)	0.3631 (5)	0.31982 (13)	0.0301 (11)	
H10	0.3628	0.3614	0.3325	0.036*	
C11	0.4784 (5)	0.2378 (5)	0.28707 (15)	0.0404 (13)	
H11	0.4224	0.1505	0.2779	0.048*	
C12	0.5939 (5)	0.2384 (6)	0.26765 (14)	0.0430 (14)	
H12	0.6174	0.1534	0.2449	0.052*	
C13	0.6743 (5)	0.3641 (5)	0.28186 (13)	0.0353 (12)	
H13	0.7542	0.3657	0.2689	0.042*	
C14	0.6403 (4)	0.4878 (5)	0.31469 (12)	0.0303 (11)	
H14	0.6978	0.5727	0.3242	0.036*	
O1	0.3123 (3)	0.9043 (3)	0.43001 (9)	0.0297 (7)	
O2	0.3534 (3)	0.6375 (3)	0.36550 (9)	0.0270 (7)	
H2o	0.328 (4)	0.722 (5)	0.3816 (13)	0.032*	
O3	0.4425 (3)	0.4516 (3)	0.44161 (9)	0.0280 (8)	
H3o	0.375 (4)	0.464 (5)	0.4309 (14)	0.034*	

### (I) (±)-(3aR,4S,5S,6aS)-4-Methyl-5-phenylhexahydro-1H-cyclopenta[c]furan-4,5-diol Atomic displacement parameters (Å^2^)

**Table:**

	*U*^11^	*U*^22^	*U*^33^	*U*^12^	*U*^13^	*U*^23^
C1	0.016 (3)	0.031 (2)	0.0249 (19)	0.0010 (19)	−0.0017 (19)	−0.0017 (17)
C2	0.018 (3)	0.018 (2)	0.039 (2)	−0.0007 (19)	0.000 (2)	−0.0036 (17)
C3	0.031 (3)	0.022 (2)	0.033 (2)	0.000 (2)	0.006 (2)	0.0035 (17)
C4	0.031 (3)	0.028 (2)	0.029 (2)	0.008 (2)	0.007 (2)	0.0026 (18)
C5	0.023 (3)	0.026 (2)	0.027 (2)	0.0007 (19)	0.0027 (19)	0.0022 (17)
C6	0.018 (3)	0.021 (2)	0.028 (2)	−0.0039 (19)	−0.0048 (19)	0.0027 (17)
C7	0.018 (3)	0.023 (2)	0.0213 (18)	0.0007 (18)	0.0035 (18)	0.0011 (16)
C8	0.026 (3)	0.028 (2)	0.023 (2)	0.002 (2)	−0.0040 (19)	−0.0017 (16)
C9	0.035 (3)	0.022 (2)	0.0139 (18)	0.004 (2)	−0.0063 (19)	0.0021 (16)
C10	0.036 (3)	0.028 (2)	0.026 (2)	0.002 (2)	−0.008 (2)	0.0031 (19)
C11	0.058 (4)	0.025 (3)	0.039 (2)	0.004 (3)	−0.023 (3)	−0.004 (2)
C12	0.067 (4)	0.033 (3)	0.029 (2)	0.028 (3)	−0.013 (3)	−0.011 (2)
C13	0.051 (4)	0.032 (3)	0.024 (2)	0.017 (2)	−0.002 (2)	0.0019 (19)
C14	0.042 (3)	0.028 (2)	0.0209 (19)	0.009 (2)	0.003 (2)	0.0024 (18)
O1	0.0226 (19)	0.0266 (15)	0.0399 (16)	0.0066 (13)	0.0054 (14)	0.0074 (12)
O2	0.023 (2)	0.0290 (17)	0.0293 (15)	0.0018 (14)	−0.0068 (14)	0.0002 (12)
O3	0.027 (2)	0.0289 (16)	0.0283 (15)	−0.0050 (15)	−0.0050 (14)	0.0076 (12)

### (I) (±)-(3aR,4S,5S,6aS)-4-Methyl-5-phenylhexahydro-1H-cyclopenta[c]furan-4,5-diol Geometric parameters (Å, º)

**Table:**

C1—C4	1.528 (5)	C7—O3	1.417 (4)
C1—C7	1.550 (5)	C7—C8	1.517 (5)
C1—C2	1.565 (5)	C8—H8A	0.9800
C1—H1	1.0000	C8—H8B	0.9800
C2—C3	1.530 (5)	C8—H8C	0.9800
C2—C5	1.543 (5)	C9—C14	1.390 (5)
C2—H2	1.0000	C9—C10	1.399 (5)
C3—O1	1.444 (4)	C10—C11	1.388 (5)
C3—H3A	0.9900	C10—H10	0.9500
C3—H3B	0.9900	C11—C12	1.381 (6)
C4—O1	1.441 (4)	C11—H11	0.9500
C4—H4A	0.9900	C12—C13	1.373 (6)
C4—H4B	0.9900	C12—H12	0.9500
C5—C6	1.519 (5)	C13—C14	1.377 (5)
C5—H5A	0.9900	C13—H13	0.9500
C5—H5B	0.9900	C14—H14	0.9500
C6—O2	1.442 (5)	O2—H2o	0.84 (4)
C6—C9	1.511 (5)	O3—H3o	0.80 (4)
C6—C7	1.559 (5)		
			
C4—C1—C7	116.4 (3)	C5—C6—C7	102.9 (3)
C4—C1—C2	103.1 (3)	O3—C7—C8	105.0 (3)
C7—C1—C2	105.9 (3)	O3—C7—C1	114.3 (3)
C4—C1—H1	110.4	C8—C7—C1	108.4 (3)
C7—C1—H1	110.4	O3—C7—C6	112.2 (3)
C2—C1—H1	110.4	C8—C7—C6	112.8 (3)
C3—C2—C5	114.7 (3)	C1—C7—C6	104.2 (3)
C3—C2—C1	103.9 (3)	C7—C8—H8A	109.5
C5—C2—C1	105.6 (3)	C7—C8—H8B	109.5
C3—C2—H2	110.8	H8A—C8—H8B	109.5
C5—C2—H2	110.8	C7—C8—H8C	109.5
C1—C2—H2	110.8	H8A—C8—H8C	109.5
O1—C3—C2	104.9 (3)	H8B—C8—H8C	109.5
O1—C3—H3A	110.8	C14—C9—C10	117.1 (4)
C2—C3—H3A	110.8	C14—C9—C6	122.7 (4)
O1—C3—H3B	110.8	C10—C9—C6	120.2 (4)
C2—C3—H3B	110.8	C11—C10—C9	120.7 (4)
H3A—C3—H3B	108.8	C11—C10—H10	119.6
O1—C4—C1	106.5 (3)	C9—C10—H10	119.6
O1—C4—H4A	110.4	C12—C11—C10	120.8 (4)
C1—C4—H4A	110.4	C12—C11—H11	119.6
O1—C4—H4B	110.4	C10—C11—H11	119.6
C1—C4—H4B	110.4	C13—C12—C11	118.8 (4)
H4A—C4—H4B	108.6	C13—C12—H12	120.6
C6—C5—C2	106.8 (3)	C11—C12—H12	120.6
C6—C5—H5A	110.4	C12—C13—C14	120.7 (5)
C2—C5—H5A	110.4	C12—C13—H13	119.7
C6—C5—H5B	110.4	C14—C13—H13	119.7
C2—C5—H5B	110.4	C13—C14—C9	121.8 (4)
H5A—C5—H5B	108.6	C13—C14—H14	119.1
O2—C6—C9	105.8 (3)	C9—C14—H14	119.1
O2—C6—C5	109.6 (3)	C4—O1—C3	104.6 (3)
C9—C6—C5	116.3 (3)	C6—O2—H2o	109 (3)
O2—C6—C7	107.5 (3)	C7—O3—H3o	107 (3)
C9—C6—C7	114.5 (3)		
			
C4—C1—C2—C3	3.5 (4)	O2—C6—C7—C8	164.9 (3)
C7—C1—C2—C3	126.2 (3)	C9—C6—C7—C8	47.6 (4)
C4—C1—C2—C5	−117.5 (3)	C5—C6—C7—C8	−79.4 (4)
C7—C1—C2—C5	5.2 (4)	O2—C6—C7—C1	−77.7 (3)
C5—C2—C3—O1	87.8 (4)	C9—C6—C7—C1	165.0 (3)
C1—C2—C3—O1	−26.9 (4)	C5—C6—C7—C1	38.0 (4)
C7—C1—C4—O1	−94.2 (3)	O2—C6—C9—C14	155.7 (3)
C2—C1—C4—O1	21.2 (4)	C5—C6—C9—C14	33.8 (5)
C3—C2—C5—C6	−94.9 (4)	C7—C6—C9—C14	−86.1 (4)
C1—C2—C5—C6	18.9 (4)	O2—C6—C9—C10	−23.8 (4)
C2—C5—C6—O2	78.9 (4)	C5—C6—C9—C10	−145.7 (3)
C2—C5—C6—C9	−161.2 (3)	C7—C6—C9—C10	94.4 (4)
C2—C5—C6—C7	−35.2 (4)	C14—C9—C10—C11	−0.2 (5)
C4—C1—C7—O3	−35.5 (4)	C6—C9—C10—C11	179.4 (3)
C2—C1—C7—O3	−149.4 (3)	C9—C10—C11—C12	−0.7 (6)
C4—C1—C7—C8	−152.3 (3)	C10—C11—C12—C13	1.0 (6)
C2—C1—C7—C8	93.9 (3)	C11—C12—C13—C14	−0.3 (6)
C4—C1—C7—C6	87.3 (4)	C12—C13—C14—C9	−0.6 (6)
C2—C1—C7—C6	−26.5 (4)	C10—C9—C14—C13	0.8 (5)
O2—C6—C7—O3	46.5 (4)	C6—C9—C14—C13	−178.7 (3)
C9—C6—C7—O3	−70.7 (4)	C1—C4—O1—C3	−39.4 (4)
C5—C6—C7—O3	162.2 (3)	C2—C3—O1—C4	41.3 (3)

### (I) (±)-(3aR,4S,5S,6aS)-4-Methyl-5-phenylhexahydro-1H-cyclopenta[c]furan-4,5-diol Hydrogen-bond geometry (Å, º)

**Table:**

*D*—H···*A*	*D*—H	H···*A*	*D*···*A*	*D*—H···*A*
O2—H2*o*···O1	0.84 (4)	1.96 (4)	2.776 (4)	163 (4)
O3—H3*o*···O1^i^	0.80 (4)	2.11 (4)	2.844 (4)	151 (4)
O3—H3*o*···O2	0.80 (4)	2.28 (4)	2.744 (3)	118 (4)
C10—H10···O2	0.95	2.33	2.667 (5)	100

Symmetry code: (i) −*x*+1/2, *y*−1/2, *z*.

### (II) (±)-(3aR,4S,5S,6aS)-4-Benzyloxy-4-methyl-5-phenylhexahydro-1H-cyclopenta[c]furan-5-ol Crystal data

**Table:**

C_21_H_24_O_3_	*F*(000) = 696
*M**_r_* = 324.40	*D*_x_ = 1.271 Mg m^−^^3^
Monoclinic, *P*2_1_/*c*	Mo *K*α radiation, λ = 0.71073 Å
*a* = 12.8872 (3) Å	Cell parameters from 3991 reflections
*b* = 19.3544 (6) Å	θ = 2.9–27.5°
*c* = 6.8046 (1) Å	µ = 0.08 mm^−^^1^
β = 92.3907 (16)°	*T* = 120 K
*V* = 1695.75 (7) Å^3^	Block, colourless
*Z* = 4	0.14 × 0.10 × 0.04 mm

### (II) (±)-(3aR,4S,5S,6aS)-4-Benzyloxy-4-methyl-5-phenylhexahydro-1H-cyclopenta[c]furan-5-ol Data collection

**Table:**

Nonius KappaCCD diffractometer	2834 reflections with *I* > 2σ(*I*)
Radiation source: fine-focus sealed tube	*R*_int_ = 0.091
Graphite monochromator	θ_max_ = 27.6°, θ_min_ = 3.2°
ω scans	*h* = −16→16
28187 measured reflections	*k* = −25→22
3899 independent reflections	*l* = −8→8

### (II) (±)-(3aR,4S,5S,6aS)-4-Benzyloxy-4-methyl-5-phenylhexahydro-1H-cyclopenta[c]furan-5-ol Refinement

**Table:**

Refinement on *F*^2^	Secondary atom site location: difference Fourier map
Least-squares matrix: full	Hydrogen site location: inferred from neighbouring sites
*R*[*F*^2^ > 2σ(*F*^2^)] = 0.053	H atoms treated by a mixture of independent and constrained refinement
*wR*(*F*^2^) = 0.132	*w* = 1/[σ^2^(*F*_o_^2^) + (0.0522*P*)^2^ + 0.6264*P*] where *P* = (*F*_o_^2^ + 2*F*_c_^2^)/3
*S* = 1.06	(Δ/σ)_max_ < 0.001
3899 reflections	Δρ_max_ = 0.30 e Å^−^^3^
222 parameters	Δρ_min_ = −0.23 e Å^−^^3^
0 restraints	Extinction correction: SHELXL97 (Sheldrick, 2008), Fc^*^=kFc[1+0.001xFc^2^λ^3^/sin(2θ)]^-1/4^
Primary atom site location: structure-invariant direct methods	Extinction coefficient: 0.015 (2)

### (II) (±)-(3aR,4S,5S,6aS)-4-Benzyloxy-4-methyl-5-phenylhexahydro-1H-cyclopenta[c]furan-5-ol Special details

**Table:**

Geometry. All esds (except the esd in the dihedral angle between two l.s. planes) are estimated using the full covariance matrix. The cell esds are taken into account individually in the estimation of esds in distances, angles and torsion angles; correlations between esds in cell parameters are only used when they are defined by crystal symmetry. An approximate (isotropic) treatment of cell esds is used for estimating esds involving l.s. planes.
Refinement. Refinement of F^2^ against ALL reflections. The weighted R-factor wR and goodness of fit S are based on F^2^, conventional R-factors R are based on F, with F set to zero for negative F^2^. The threshold expression of F^2^ > 2sigma(F^2^) is used only for calculating R-factors(gt) etc. and is not relevant to the choice of reflections for refinement. R-factors based on F^2^ are statistically about twice as large as those based on F, and R- factors based on ALL data will be even larger.

### (II) (±)-(3aR,4S,5S,6aS)-4-Benzyloxy-4-methyl-5-phenylhexahydro-1H-cyclopenta[c]furan-5-ol Fractional atomic coordinates and isotropic or equivalent isotropic displacement parameters (Å^2^)

**Table:**

	*x*	*y*	*z*	*U*_iso_*/*U*_eq_	
C1	0.30147 (13)	0.58494 (9)	0.3553 (2)	0.0281 (4)	
H1	0.2965	0.5555	0.2345	0.034*	
C2	0.29204 (13)	0.66286 (9)	0.2994 (2)	0.0287 (4)	
H2	0.2808	0.6687	0.1541	0.034*	
C3	0.39707 (14)	0.69274 (10)	0.3697 (3)	0.0363 (4)	
H3A	0.4456	0.6937	0.2608	0.044*	
H3B	0.3887	0.7404	0.4198	0.044*	
C4	0.40989 (14)	0.57980 (10)	0.4536 (3)	0.0350 (4)	
H4A	0.4103	0.5463	0.5636	0.042*	
H4B	0.4607	0.5644	0.3576	0.042*	
C5	0.19910 (13)	0.69099 (9)	0.4091 (2)	0.0269 (4)	
H5A	0.2134	0.7385	0.4569	0.032*	
H5B	0.1360	0.6919	0.3212	0.032*	
C6	0.18412 (12)	0.64195 (8)	0.5828 (2)	0.0225 (4)	
C7	0.20787 (13)	0.57048 (9)	0.4881 (2)	0.0242 (4)	
C8	0.11588 (14)	0.54513 (9)	0.3603 (2)	0.0300 (4)	
H8A	0.1346	0.5021	0.2948	0.045*	
H8B	0.0972	0.5802	0.2611	0.045*	
H8C	0.0566	0.5368	0.4428	0.045*	
C9	0.07863 (12)	0.64665 (9)	0.6741 (2)	0.0237 (4)	
C10	0.05847 (13)	0.60608 (9)	0.8379 (2)	0.0284 (4)	
H10	0.1113	0.5767	0.8921	0.034*	
C11	−0.03727 (14)	0.60800 (10)	0.9227 (2)	0.0317 (4)	
H11	−0.0498	0.5796	1.0332	0.038*	
C12	−0.11497 (14)	0.65112 (10)	0.8471 (2)	0.0322 (4)	
H12	−0.1810	0.6521	0.9042	0.039*	
C13	−0.09541 (14)	0.69271 (10)	0.6879 (3)	0.0321 (4)	
H13	−0.1479	0.7231	0.6370	0.039*	
C14	0.00014 (13)	0.69059 (9)	0.6015 (2)	0.0280 (4)	
H14	0.0123	0.7194	0.4917	0.034*	
C15	0.24848 (15)	0.45363 (9)	0.5913 (2)	0.0311 (4)	
H15A	0.2899	0.4514	0.4721	0.037*	
H15B	0.1807	0.4310	0.5618	0.037*	
C16	0.30523 (13)	0.41712 (9)	0.7601 (2)	0.0261 (4)	
C17	0.31224 (14)	0.34524 (9)	0.7586 (3)	0.0295 (4)	
H17	0.2798	0.3199	0.6534	0.035*	
C18	0.36607 (14)	0.31032 (10)	0.9088 (3)	0.0324 (4)	
H18	0.3709	0.2614	0.9053	0.039*	
C19	0.41288 (14)	0.34674 (10)	1.0645 (3)	0.0329 (4)	
H19	0.4494	0.3230	1.1680	0.039*	
C20	0.40569 (14)	0.41784 (10)	1.0669 (3)	0.0335 (4)	
H20	0.4376	0.4430	1.1731	0.040*	
C21	0.35245 (14)	0.45329 (9)	0.9163 (2)	0.0297 (4)	
H22	0.3483	0.5023	0.9200	0.036*	
O1	0.43553 (9)	0.64763 (7)	0.52482 (18)	0.0359 (3)	
O2	0.25800 (9)	0.65642 (6)	0.74180 (16)	0.0258 (3)	
H2o	0.3204 (16)	0.6532 (10)	0.697 (3)	0.031*	
O3	0.23287 (9)	0.52361 (6)	0.64562 (15)	0.0276 (3)	

### (II) (±)-(3aR,4S,5S,6aS)-4-Benzyloxy-4-methyl-5-phenylhexahydro-1H-cyclopenta[c]furan-5-ol Atomic displacement parameters (Å^2^)

**Table:**

	*U*^11^	*U*^22^	*U*^33^	*U*^12^	*U*^13^	*U*^23^
C1	0.0325 (9)	0.0287 (10)	0.0232 (8)	0.0023 (7)	0.0020 (7)	−0.0027 (7)
C2	0.0291 (9)	0.0303 (10)	0.0269 (8)	0.0007 (7)	0.0043 (7)	0.0043 (7)
C3	0.0324 (10)	0.0370 (11)	0.0402 (10)	−0.0025 (8)	0.0085 (8)	0.0044 (8)
C4	0.0307 (9)	0.0360 (11)	0.0383 (10)	0.0055 (8)	0.0045 (8)	0.0030 (8)
C5	0.0284 (9)	0.0246 (9)	0.0278 (8)	−0.0002 (7)	0.0016 (7)	0.0056 (7)
C6	0.0243 (8)	0.0220 (8)	0.0210 (7)	0.0003 (6)	−0.0027 (6)	0.0007 (6)
C7	0.0309 (9)	0.0220 (8)	0.0194 (7)	0.0006 (7)	−0.0006 (6)	0.0015 (6)
C8	0.0362 (10)	0.0307 (10)	0.0229 (8)	−0.0052 (8)	−0.0004 (7)	−0.0015 (7)
C9	0.0264 (8)	0.0221 (8)	0.0225 (7)	−0.0017 (6)	−0.0006 (6)	−0.0036 (6)
C10	0.0305 (9)	0.0300 (10)	0.0247 (8)	0.0007 (7)	−0.0008 (7)	0.0006 (7)
C11	0.0319 (9)	0.0396 (11)	0.0238 (8)	−0.0052 (8)	0.0025 (7)	−0.0006 (7)
C12	0.0249 (9)	0.0420 (11)	0.0298 (9)	−0.0039 (8)	0.0042 (7)	−0.0099 (8)
C13	0.0275 (9)	0.0357 (10)	0.0326 (9)	0.0038 (8)	−0.0032 (7)	−0.0038 (8)
C14	0.0288 (9)	0.0292 (9)	0.0260 (8)	0.0016 (7)	−0.0010 (7)	0.0001 (7)
C15	0.0464 (11)	0.0221 (9)	0.0245 (8)	0.0020 (8)	−0.0005 (8)	−0.0023 (7)
C16	0.0291 (9)	0.0247 (9)	0.0250 (8)	0.0002 (7)	0.0060 (7)	−0.0003 (7)
C17	0.0331 (9)	0.0262 (9)	0.0297 (9)	0.0005 (7)	0.0073 (7)	−0.0011 (7)
C18	0.0356 (10)	0.0242 (9)	0.0382 (10)	0.0051 (7)	0.0128 (8)	0.0035 (8)
C19	0.0298 (9)	0.0370 (11)	0.0322 (9)	0.0075 (8)	0.0046 (7)	0.0082 (8)
C20	0.0345 (10)	0.0360 (11)	0.0297 (9)	0.0018 (8)	−0.0007 (7)	−0.0020 (8)
C21	0.0384 (10)	0.0241 (9)	0.0266 (8)	0.0011 (7)	0.0007 (7)	−0.0015 (7)
O1	0.0288 (7)	0.0435 (8)	0.0354 (7)	−0.0024 (6)	0.0003 (5)	−0.0020 (6)
O2	0.0240 (6)	0.0287 (7)	0.0245 (6)	0.0000 (5)	−0.0012 (5)	−0.0052 (5)
O3	0.0421 (7)	0.0206 (6)	0.0199 (5)	0.0046 (5)	−0.0009 (5)	0.0002 (4)

### (II) (±)-(3aR,4S,5S,6aS)-4-Benzyloxy-4-methyl-5-phenylhexahydro-1H-cyclopenta[c]furan-5-ol Geometric parameters (Å, º)

**Table:**

C1—C4	1.527 (2)	C10—C11	1.384 (2)
C1—C2	1.559 (2)	C10—H10	0.9500
C1—C7	1.562 (2)	C11—C12	1.386 (3)
C1—H1	1.0000	C11—H11	0.9500
C2—C3	1.530 (3)	C12—C13	1.381 (3)
C2—C5	1.537 (2)	C12—H12	0.9500
C2—H2	1.0000	C13—C14	1.387 (2)
C3—O1	1.441 (2)	C13—H13	0.9500
C3—H3A	0.9900	C14—H14	0.9500
C3—H3B	0.9900	C15—O3	1.421 (2)
C4—O1	1.433 (2)	C15—C16	1.511 (2)
C4—H4A	0.9900	C15—H15A	0.9900
C4—H4B	0.9900	C15—H15B	0.9900
C5—C6	1.534 (2)	C16—C21	1.391 (2)
C5—H5A	0.9900	C16—C17	1.394 (2)
C5—H5B	0.9900	C17—C18	1.387 (3)
C6—O2	1.4387 (19)	C17—H17	0.9500
C6—C9	1.521 (2)	C18—C19	1.389 (3)
C6—C7	1.562 (2)	C18—H18	0.9500
C7—O3	1.4305 (19)	C19—C20	1.379 (3)
C7—C8	1.522 (2)	C19—H19	0.9500
C8—H8A	0.9800	C20—C21	1.391 (2)
C8—H8B	0.9800	C20—H20	0.9500
C8—H8C	0.9800	C21—H22	0.9500
C9—C14	1.396 (2)	O2—H2o	0.87 (2)
C9—C10	1.397 (2)		
			
C4—C1—C2	103.32 (14)	H8B—C8—H8C	109.5
C4—C1—C7	116.71 (14)	C14—C9—C10	117.94 (15)
C2—C1—C7	105.08 (13)	C14—C9—C6	122.61 (15)
C4—C1—H1	110.4	C10—C9—C6	119.45 (14)
C2—C1—H1	110.4	C11—C10—C9	121.08 (16)
C7—C1—H1	110.4	C11—C10—H10	119.5
C3—C2—C5	114.28 (15)	C9—C10—H10	119.5
C3—C2—C1	103.32 (14)	C10—C11—C12	120.28 (17)
C5—C2—C1	106.17 (13)	C10—C11—H11	119.9
C3—C2—H2	110.9	C12—C11—H11	119.9
C5—C2—H2	110.9	C13—C12—C11	119.30 (16)
C1—C2—H2	110.9	C13—C12—H12	120.3
O1—C3—C2	105.82 (14)	C11—C12—H12	120.3
O1—C3—H3A	110.6	C12—C13—C14	120.61 (17)
C2—C3—H3A	110.6	C12—C13—H13	119.7
O1—C3—H3B	110.6	C14—C13—H13	119.7
C2—C3—H3B	110.6	C13—C14—C9	120.75 (16)
H3A—C3—H3B	108.7	C13—C14—H14	119.6
O1—C4—C1	106.40 (14)	C9—C14—H14	119.6
O1—C4—H4A	110.4	O3—C15—C16	108.51 (13)
C1—C4—H4A	110.4	O3—C15—H15A	110.0
O1—C4—H4B	110.4	C16—C15—H15A	110.0
C1—C4—H4B	110.4	O3—C15—H15B	110.0
H4A—C4—H4B	108.6	C16—C15—H15B	110.0
C6—C5—C2	106.29 (13)	H15A—C15—H15B	108.4
C6—C5—H5A	110.5	C21—C16—C17	118.78 (16)
C2—C5—H5A	110.5	C21—C16—C15	121.85 (15)
C6—C5—H5B	110.5	C17—C16—C15	119.36 (15)
C2—C5—H5B	110.5	C18—C17—C16	120.74 (17)
H5A—C5—H5B	108.7	C18—C17—H17	119.6
O2—C6—C9	104.83 (12)	C16—C17—H17	119.6
O2—C6—C5	111.00 (13)	C17—C18—C19	120.17 (17)
C9—C6—C5	114.90 (13)	C17—C18—H18	119.9
O2—C6—C7	110.35 (12)	C19—C18—H18	119.9
C9—C6—C7	114.54 (13)	C20—C19—C18	119.24 (16)
C5—C6—C7	101.38 (12)	C20—C19—H19	120.4
O3—C7—C8	111.68 (13)	C18—C19—H19	120.4
O3—C7—C6	107.13 (12)	C19—C20—C21	120.97 (17)
C8—C7—C6	111.09 (14)	C19—C20—H20	119.5
O3—C7—C1	113.06 (13)	C21—C20—H20	119.5
C8—C7—C1	109.21 (13)	C20—C21—C16	120.10 (16)
C6—C7—C1	104.43 (13)	C20—C21—H22	120.0
C7—C8—H8A	109.5	C16—C21—H22	120.0
C7—C8—H8B	109.5	C4—O1—C3	103.87 (13)
H8A—C8—H8B	109.5	C6—O2—H2o	108.4 (12)
C7—C8—H8C	109.5	C15—O3—C7	116.08 (12)
H8A—C8—H8C	109.5		
			
C4—C1—C2—C3	0.84 (16)	C5—C6—C9—C14	2.0 (2)
C7—C1—C2—C3	123.68 (14)	C7—C6—C9—C14	−114.80 (17)
C4—C1—C2—C5	−119.73 (14)	O2—C6—C9—C10	−55.45 (18)
C7—C1—C2—C5	3.10 (17)	C5—C6—C9—C10	−177.55 (15)
C5—C2—C3—O1	89.86 (17)	C7—C6—C9—C10	65.64 (19)
C1—C2—C3—O1	−25.02 (17)	C14—C9—C10—C11	1.7 (2)
C2—C1—C4—O1	23.80 (16)	C6—C9—C10—C11	−178.76 (15)
C7—C1—C4—O1	−90.94 (17)	C9—C10—C11—C12	−0.7 (3)
C3—C2—C5—C6	−91.02 (17)	C10—C11—C12—C13	−0.8 (3)
C1—C2—C5—C6	22.18 (17)	C11—C12—C13—C14	1.3 (3)
C2—C5—C6—O2	79.01 (16)	C12—C13—C14—C9	−0.3 (3)
C2—C5—C6—C9	−162.29 (13)	C10—C9—C14—C13	−1.2 (2)
C2—C5—C6—C7	−38.20 (16)	C6—C9—C14—C13	179.27 (15)
O2—C6—C7—O3	42.19 (17)	O3—C15—C16—C21	−13.3 (2)
C9—C6—C7—O3	−75.80 (16)	O3—C15—C16—C17	167.77 (15)
C5—C6—C7—O3	159.87 (12)	C21—C16—C17—C18	−0.6 (2)
O2—C6—C7—C8	164.41 (13)	C15—C16—C17—C18	178.35 (16)
C9—C6—C7—C8	46.42 (17)	C16—C17—C18—C19	0.7 (3)
C5—C6—C7—C8	−77.91 (15)	C17—C18—C19—C20	−0.4 (3)
O2—C6—C7—C1	−77.98 (15)	C18—C19—C20—C21	0.0 (3)
C9—C6—C7—C1	164.03 (13)	C19—C20—C21—C16	0.1 (3)
C5—C6—C7—C1	39.70 (15)	C17—C16—C21—C20	0.2 (2)
C4—C1—C7—O3	−29.0 (2)	C15—C16—C21—C20	−178.73 (16)
C2—C1—C7—O3	−142.74 (13)	C1—C4—O1—C3	−40.50 (17)
C4—C1—C7—C8	−153.99 (15)	C2—C3—O1—C4	40.92 (17)
C2—C1—C7—C8	92.26 (16)	C16—C15—O3—C7	162.70 (13)
C4—C1—C7—C6	87.11 (17)	C8—C7—O3—C15	52.94 (19)
C2—C1—C7—C6	−26.63 (16)	C6—C7—O3—C15	174.79 (14)
O2—C6—C9—C14	124.12 (16)	C1—C7—O3—C15	−70.71 (18)

### (II) (±)-(3aR,4S,5S,6aS)-4-Benzyloxy-4-methyl-5-phenylhexahydro-1H-cyclopenta[c]furan-5-ol Hydrogen-bond geometry (Å, º)

Cg4 is the centroid of the C16–C21 ring.

**Table:**

*D*—H···*A*	*D*—H	H···*A*	*D*···*A*	*D*—H···*A*
O2—H2*o*···O1	0.87 (2)	1.93 (2)	2.7794 (17)	162.9 (18)
C10—H10···O3	0.95	2.56	3.091 (2)	116
C5—H5*A*···O2^i^	0.99	2.58	3.266 (2)	126
C19—H19···O1^ii^	0.95	2.58	3.344 (2)	138
C12—H12···*Cg*4^iii^	0.95	2.74	3.6619 (19)	165

Symmetry codes: (i) *x*, −*y*+3/2, *z*−1/2; (ii) −*x*+1, −*y*+1, −*z*+2; (iii) −*x*, −*y*+1, −*z*+2.

### (III) (±)-(1aR,1bS,4aR,5S,5aR)-5-Benzyloxy-5-methyl-5a-phenylhexahydro-2H-oxireno[2',3':3,4]cyclopenta[1,2-c]furan Crystal data

**Table:**

C_21_H_22_O_3_	*D*_x_ = 1.291 Mg m^−^^3^
*M**_r_* = 322.39	Mo *K*α radiation, λ = 0.71073 Å
Orthorhombic, *P*2_1_2_1_2_1_	Cell parameters from 2191 reflections
*a* = 5.6392 (2) Å	θ = 2.9–27.5°
*b* = 11.0427 (5) Å	µ = 0.09 mm^−^^1^
*c* = 26.6311 (13) Å	*T* = 120 K
*V* = 1658.37 (13) Å^3^	Block, colourless
*Z* = 4	0.34 × 0.14 × 0.04 mm
*F*(000) = 688	

### (III) (±)-(1aR,1bS,4aR,5S,5aR)-5-Benzyloxy-5-methyl-5a-phenylhexahydro-2H-oxireno[2',3':3,4]cyclopenta[1,2-c]furan Data collection

**Table:**

Nonius KappaCCD diffractometer	1867 reflections with *I* > 2σ(*I*)
Radiation source: fine-focus sealed tube	*R*_int_ = 0.073
Graphite monochromator	θ_max_ = 27.5°, θ_min_ = 2.9°
ω scans	*h* = −7→7
12562 measured reflections	*k* = −14→10
2221 independent reflections	*l* = −33→34

### (III) (±)-(1aR,1bS,4aR,5S,5aR)-5-Benzyloxy-5-methyl-5a-phenylhexahydro-2H-oxireno[2',3':3,4]cyclopenta[1,2-c]furan Refinement

**Table:**

Refinement on *F*^2^	Secondary atom site location: difference Fourier map
Least-squares matrix: full	Hydrogen site location: inferred from neighbouring sites
*R*[*F*^2^ > 2σ(*F*^2^)] = 0.123	H-atom parameters constrained
*wR*(*F*^2^) = 0.279	*w* = 1/[σ^2^(*F*_o_^2^) + (0.*P*)^2^ + 10.5966*P*] where *P* = (*F*_o_^2^ + 2*F*_c_^2^)/3
*S* = 1.17	(Δ/σ)_max_ = 0.001
2221 reflections	Δρ_max_ = 0.40 e Å^−^^3^
190 parameters	Δρ_min_ = −0.44 e Å^−^^3^
0 restraints	Extinction correction: SHELXL97 (Sheldrick, 2008), Fc^*^=kFc[1+0.001xFc^2^λ^3^/sin(2θ)]^-1/4^
Primary atom site location: structure-invariant direct methods	Extinction coefficient: 0.027 (5)

### (III) (±)-(1aR,1bS,4aR,5S,5aR)-5-Benzyloxy-5-methyl-5a-phenylhexahydro-2H-oxireno[2',3':3,4]cyclopenta[1,2-c]furan Special details

**Table:**

Geometry. All esds (except the esd in the dihedral angle between two l.s. planes) are estimated using the full covariance matrix. The cell esds are taken into account individually in the estimation of esds in distances, angles and torsion angles; correlations between esds in cell parameters are only used when they are defined by crystal symmetry. An approximate (isotropic) treatment of cell esds is used for estimating esds involving l.s. planes.
Refinement. Refinement of F^2^ against ALL reflections. The weighted R-factor wR and goodness of fit S are based on F^2^, conventional R-factors R are based on F, with F set to zero for negative F^2^. The threshold expression of F^2^ > 2sigma(F^2^) is used only for calculating R-factors(gt) etc. and is not relevant to the choice of reflections for refinement. R-factors based on F^2^ are statistically about twice as large as those based on F, and R- factors based on ALL data will be even larger.

### (III) (±)-(1aR,1bS,4aR,5S,5aR)-5-Benzyloxy-5-methyl-5a-phenylhexahydro-2H-oxireno[2',3':3,4]cyclopenta[1,2-c]furan Fractional atomic coordinates and isotropic or equivalent isotropic displacement parameters (Å^2^)

**Table:**

	*x*	*y*	*z*	*U*_iso_*/*U*_eq_	Occ. (<1)
C1	0.6868 (17)	0.6626 (7)	0.1385 (3)	0.032 (2)	
H1	0.8255	0.6533	0.1615	0.038*	
C2	0.7362 (19)	0.7660 (7)	0.1007 (3)	0.035 (2)	
H2	0.9101	0.7763	0.0947	0.042*	
C3	0.631 (2)	0.8762 (7)	0.1259 (4)	0.045 (3)	
H3A	0.7478	0.9148	0.1484	0.054*	
H3B	0.5793	0.9363	0.1005	0.054*	
C4	0.471 (2)	0.7092 (8)	0.1684 (3)	0.041 (2)	
H4A	0.3289	0.6596	0.1608	0.049*	
H4B	0.5024	0.7042	0.2049	0.049*	
C5	0.6092 (14)	0.7235 (7)	0.0536 (3)	0.0232 (16)	
H5	0.6490	0.7608	0.0204	0.028*	
C6	0.5563 (14)	0.5938 (7)	0.0566 (3)	0.0247 (17)	
C7	0.6606 (15)	0.5476 (7)	0.1061 (3)	0.0230 (16)	
C8	0.9005 (16)	0.4920 (7)	0.0942 (3)	0.0301 (18)	
H8A	0.9849	0.4746	0.1255	0.045*	
H8B	0.9937	0.5490	0.0740	0.045*	
H8C	0.8778	0.4167	0.0753	0.045*	
C9	0.5260 (13)	0.5131 (7)	0.0124 (3)	0.0210 (15)	
C10	0.3788 (15)	0.4124 (7)	0.0148 (3)	0.0298 (18)	
H10	0.2965	0.3951	0.0451	0.036*	
C11	0.3498 (17)	0.3360 (8)	−0.0267 (3)	0.036 (2)	
H11	0.2495	0.2671	−0.0245	0.043*	
C12	0.4673 (19)	0.3614 (8)	−0.0704 (3)	0.038 (2)	
H12	0.4526	0.3088	−0.0985	0.046*	
C13	0.606 (2)	0.4624 (9)	−0.0735 (3)	0.060 (4)	
H13	0.6799	0.4824	−0.1044	0.072*	
C14	0.639 (2)	0.5365 (9)	−0.0319 (3)	0.046 (3)	
H14	0.7420	0.6043	−0.0343	0.055*	
C15	0.5820 (19)	0.4029 (9)	0.1723 (3)	0.042 (2)	
H15A	0.7256	0.3557	0.1638	0.050*	
H15B	0.6280	0.4654	0.1972	0.050*	
C17A	0.232 (3)	0.3698 (9)	0.2235 (6)	0.030 (6)*	0.54 (3)
H17A	0.2215	0.4538	0.2309	0.035*	0.54 (3)
C18A	0.062 (2)	0.2904 (12)	0.2423 (5)	0.038 (5)*	0.54 (3)
H18A	−0.0637	0.3201	0.2625	0.045*	0.54 (3)
C19A	0.077 (3)	0.1675 (12)	0.2315 (5)	0.034 (4)*	0.54 (3)
H19A	−0.0389	0.1132	0.2443	0.041*	0.54 (3)
C20A	0.261 (3)	0.1240 (9)	0.2019 (4)	0.032 (5)*	0.54 (3)
H20A	0.2711	0.0399	0.1945	0.038*	0.54 (3)
C21A	0.431 (3)	0.2033 (11)	0.1831 (4)	0.037 (5)*	0.54 (3)
H21A	0.5564	0.1736	0.1628	0.045*	0.54 (3)
C16A	0.416 (2)	0.3262 (10)	0.1939 (6)	0.026 (5)*	0.54 (3)
C17B	0.226 (3)	0.3804 (10)	0.2286 (7)	0.035 (8)*	0.46 (3)
H17B	0.2537	0.4629	0.2369	0.043*	0.46 (3)
C18B	0.040 (3)	0.3181 (13)	0.2510 (6)	0.035 (5)*	0.46 (3)
H18B	−0.0600	0.3581	0.2746	0.042*	0.46 (3)
C19B	−0.001 (3)	0.1974 (14)	0.2390 (5)	0.030 (5)*	0.46 (3)
H19B	−0.1285	0.1549	0.2543	0.036*	0.46 (3)
C20B	0.145 (4)	0.1390 (10)	0.2046 (5)	0.034 (5)*	0.46 (3)
H20B	0.1167	0.0565	0.1963	0.041*	0.46 (3)
C21B	0.331 (4)	0.2012 (13)	0.1822 (5)	0.029 (5)*	0.46 (3)
H21B	0.4304	0.1612	0.1586	0.035*	0.46 (3)
C16B	0.372 (3)	0.3219 (13)	0.1942 (7)	0.032 (6)*	0.46 (3)
O1	0.4314 (16)	0.8325 (6)	0.1539 (2)	0.057 (2)	
O2	0.3649 (10)	0.6835 (5)	0.0638 (2)	0.0279 (13)	
O3	0.4946 (11)	0.4616 (5)	0.12770 (19)	0.0301 (13)	

### (III) (±)-(1aR,1bS,4aR,5S,5aR)-5-Benzyloxy-5-methyl-5a-phenylhexahydro-2H-oxireno[2',3':3,4]cyclopenta[1,2-c]furan Atomic displacement parameters (Å^2^)

**Table:**

	*U*^11^	*U*^22^	*U*^33^	*U*^12^	*U*^13^	*U*^23^
C1	0.043 (5)	0.025 (4)	0.027 (4)	0.011 (4)	−0.011 (4)	−0.004 (3)
C2	0.046 (6)	0.025 (4)	0.033 (4)	0.014 (4)	−0.003 (4)	−0.006 (3)
C3	0.072 (8)	0.020 (4)	0.043 (5)	0.010 (5)	−0.014 (6)	−0.003 (4)
C4	0.060 (6)	0.037 (5)	0.026 (4)	0.023 (5)	−0.007 (4)	−0.001 (4)
C5	0.018 (4)	0.027 (4)	0.025 (4)	0.002 (3)	0.003 (3)	0.003 (3)
C6	0.021 (4)	0.031 (4)	0.023 (3)	0.013 (3)	0.000 (3)	0.006 (3)
C7	0.025 (4)	0.019 (3)	0.025 (3)	0.004 (3)	−0.005 (3)	0.000 (3)
C8	0.028 (4)	0.024 (4)	0.038 (4)	0.005 (4)	−0.005 (4)	−0.005 (3)
C9	0.017 (3)	0.024 (4)	0.022 (3)	−0.003 (3)	−0.004 (3)	0.004 (3)
C10	0.017 (4)	0.026 (4)	0.046 (4)	0.000 (3)	0.012 (4)	0.000 (4)
C11	0.033 (5)	0.025 (4)	0.049 (5)	−0.003 (4)	0.004 (4)	−0.006 (4)
C12	0.058 (6)	0.028 (4)	0.028 (4)	−0.015 (5)	−0.003 (4)	−0.007 (3)
C13	0.094 (10)	0.052 (6)	0.034 (5)	−0.041 (7)	0.024 (6)	−0.014 (4)
C14	0.059 (7)	0.054 (6)	0.025 (4)	−0.041 (6)	0.013 (4)	−0.007 (4)
C15	0.042 (6)	0.051 (5)	0.032 (4)	0.005 (5)	0.001 (4)	0.020 (4)
O1	0.084 (6)	0.044 (4)	0.042 (4)	0.033 (4)	0.013 (4)	0.003 (3)
O2	0.021 (3)	0.035 (3)	0.028 (3)	0.007 (3)	0.006 (2)	0.004 (2)
O3	0.033 (3)	0.031 (3)	0.026 (3)	0.009 (3)	0.001 (3)	0.007 (2)

### (III) (±)-(1aR,1bS,4aR,5S,5aR)-5-Benzyloxy-5-methyl-5a-phenylhexahydro-2H-oxireno[2',3':3,4]cyclopenta[1,2-c]furan Geometric parameters (Å, º)

**Table:**

C1—C7	1.542 (10)	C12—H12	0.9500
C1—C4	1.545 (13)	C13—C14	1.390 (11)
C1—C2	1.547 (11)	C13—H13	0.9500
C1—H1	1.0000	C14—H14	0.9500
C2—C3	1.512 (11)	C15—C16A	1.387 (13)
C2—C5	1.519 (11)	C15—O3	1.441 (9)
C2—H2	1.0000	C15—C16B	1.596 (15)
C3—O1	1.432 (14)	C15—H15A	0.9900
C3—H3A	0.9900	C15—H15B	0.9900
C3—H3B	0.9900	C17A—C18A	1.3900
C4—O1	1.433 (10)	C17A—C16A	1.3900
C4—H4A	0.9900	C17A—H17A	0.9500
C4—H4B	0.9900	C18A—C19A	1.3900
C5—C6	1.465 (11)	C18A—H18A	0.9500
C5—O2	1.472 (9)	C19A—C20A	1.3900
C5—H5	1.0000	C19A—H19A	0.9500
C6—O2	1.477 (9)	C20A—C21A	1.3900
C6—C9	1.487 (10)	C20A—H20A	0.9500
C6—C7	1.532 (10)	C21A—C16A	1.3900
C7—O3	1.452 (10)	C21A—H21A	0.9500
C7—C8	1.519 (11)	C17B—C18B	1.3900
C8—H8A	0.9800	C17B—C16B	1.3900
C8—H8B	0.9800	C17B—H17B	0.9500
C8—H8C	0.9800	C18B—C19B	1.3900
C9—C14	1.364 (10)	C18B—H18B	0.9500
C9—C10	1.389 (10)	C19B—C20B	1.3900
C10—C11	1.399 (11)	C19B—H19B	0.9500
C10—H10	0.9500	C20B—C21B	1.3900
C11—C12	1.370 (12)	C20B—H20B	0.9500
C11—H11	0.9500	C21B—C16B	1.3900
C12—C13	1.364 (13)	C21B—H21B	0.9500
			
C7—C1—C4	119.2 (8)	C13—C12—H12	120.2
C7—C1—C2	105.2 (6)	C11—C12—H12	120.2
C4—C1—C2	103.4 (7)	C12—C13—C14	120.8 (9)
C7—C1—H1	109.5	C12—C13—H13	119.6
C4—C1—H1	109.5	C14—C13—H13	119.6
C2—C1—H1	109.5	C9—C14—C13	120.9 (8)
C3—C2—C5	115.4 (8)	C9—C14—H14	119.5
C3—C2—C1	103.6 (7)	C13—C14—H14	119.5
C5—C2—C1	102.9 (7)	C16A—C15—O3	112.6 (10)
C3—C2—H2	111.4	C16A—C15—C16B	5.6 (12)
C5—C2—H2	111.4	O3—C15—C16B	107.4 (10)
C1—C2—H2	111.4	C16A—C15—H15A	109.1
O1—C3—C2	105.6 (7)	O3—C15—H15A	109.1
O1—C3—H3A	110.6	C16B—C15—H15A	113.4
C2—C3—H3A	110.6	C16A—C15—H15B	109.1
O1—C3—H3B	110.6	O3—C15—H15B	109.1
C2—C3—H3B	110.6	C16B—C15—H15B	110.0
H3A—C3—H3B	108.8	H15A—C15—H15B	107.8
O1—C4—C1	107.4 (8)	C18A—C17A—C16A	120.0
O1—C4—H4A	110.2	C18A—C17A—H17A	120.0
C1—C4—H4A	110.2	C16A—C17A—H17A	120.0
O1—C4—H4B	110.2	C19A—C18A—C17A	120.0
C1—C4—H4B	110.2	C19A—C18A—H18A	120.0
H4A—C4—H4B	108.5	C17A—C18A—H18A	120.0
C6—C5—O2	60.4 (5)	C18A—C19A—C20A	120.0
C6—C5—C2	110.7 (7)	C18A—C19A—H19A	120.0
O2—C5—C2	112.4 (7)	C20A—C19A—H19A	120.0
C6—C5—H5	119.8	C21A—C20A—C19A	120.0
O2—C5—H5	119.8	C21A—C20A—H20A	120.0
C2—C5—H5	119.8	C19A—C20A—H20A	120.0
C5—C6—O2	60.1 (5)	C20A—C21A—C16A	120.0
C5—C6—C9	124.5 (7)	C20A—C21A—H21A	120.0
O2—C6—C9	114.9 (6)	C16A—C21A—H21A	120.0
C5—C6—C7	107.1 (7)	C15—C16A—C21A	118.0 (9)
O2—C6—C7	113.1 (6)	C15—C16A—C17A	121.9 (9)
C9—C6—C7	121.7 (6)	C21A—C16A—C17A	120.0
O3—C7—C8	113.2 (6)	C18B—C17B—C16B	120.0
O3—C7—C6	108.2 (6)	C18B—C17B—H17B	120.0
C8—C7—C6	107.3 (6)	C16B—C17B—H17B	120.0
O3—C7—C1	112.3 (6)	C19B—C18B—C17B	120.0
C8—C7—C1	111.3 (7)	C19B—C18B—H18B	120.0
C6—C7—C1	104.1 (6)	C17B—C18B—H18B	120.0
C7—C8—H8A	109.5	C18B—C19B—C20B	120.0
C7—C8—H8B	109.5	C18B—C19B—H19B	120.0
H8A—C8—H8B	109.5	C20B—C19B—H19B	120.0
C7—C8—H8C	109.5	C21B—C20B—C19B	120.0
H8A—C8—H8C	109.5	C21B—C20B—H20B	120.0
H8B—C8—H8C	109.5	C19B—C20B—H20B	120.0
C14—C9—C10	118.0 (7)	C20B—C21B—C16B	120.0
C14—C9—C6	121.1 (7)	C20B—C21B—H21B	120.0
C10—C9—C6	120.8 (7)	C16B—C21B—H21B	120.0
C9—C10—C11	121.1 (8)	C21B—C16B—C17B	120.0
C9—C10—H10	119.5	C21B—C16B—C15	125.2 (10)
C11—C10—H10	119.5	C17B—C16B—C15	114.8 (10)
C12—C11—C10	119.4 (8)	C3—O1—C4	109.9 (8)
C12—C11—H11	120.3	C5—O2—C6	59.5 (5)
C10—C11—H11	120.3	C15—O3—C7	113.6 (7)
C13—C12—C11	119.6 (8)		
			
C7—C1—C2—C3	149.9 (8)	C9—C10—C11—C12	−0.4 (14)
C4—C1—C2—C3	24.2 (10)	C10—C11—C12—C13	−1.8 (15)
C7—C1—C2—C5	29.3 (9)	C11—C12—C13—C14	3.5 (18)
C4—C1—C2—C5	−96.4 (8)	C10—C9—C14—C13	0.7 (16)
C5—C2—C3—O1	79.5 (9)	C6—C9—C14—C13	−178.4 (10)
C1—C2—C3—O1	−32.2 (10)	C12—C13—C14—C9	−3.0 (19)
C7—C1—C4—O1	−124.4 (8)	C16A—C17A—C18A—C19A	0.0
C2—C1—C4—O1	−8.2 (9)	C17A—C18A—C19A—C20A	0.0
C3—C2—C5—C6	−128.9 (8)	C18A—C19A—C20A—C21A	0.0
C1—C2—C5—C6	−16.8 (9)	C19A—C20A—C21A—C16A	0.0
C3—C2—C5—O2	−63.5 (10)	O3—C15—C16A—C21A	97.6 (11)
C1—C2—C5—O2	48.6 (8)	C16B—C15—C16A—C21A	118 (11)
C2—C5—C6—O2	104.7 (7)	O3—C15—C16A—C17A	−79.3 (11)
O2—C5—C6—C9	101.2 (8)	C16B—C15—C16A—C17A	−59 (11)
C2—C5—C6—C9	−154.1 (8)	C20A—C21A—C16A—C15	−176.9 (13)
O2—C5—C6—C7	−107.2 (6)	C20A—C21A—C16A—C17A	0.0
C2—C5—C6—C7	−2.5 (9)	C18A—C17A—C16A—C15	176.8 (13)
C5—C6—C7—O3	140.5 (6)	C18A—C17A—C16A—C21A	0.0
O2—C6—C7—O3	76.3 (8)	C16B—C17B—C18B—C19B	0.0
C9—C6—C7—O3	−67.0 (9)	C17B—C18B—C19B—C20B	0.0
C5—C6—C7—C8	−97.2 (7)	C18B—C19B—C20B—C21B	0.0
O2—C6—C7—C8	−161.3 (6)	C19B—C20B—C21B—C16B	0.0
C9—C6—C7—C8	55.4 (9)	C20B—C21B—C16B—C17B	0.0
C5—C6—C7—C1	20.9 (8)	C20B—C21B—C16B—C15	178.5 (15)
O2—C6—C7—C1	−43.2 (9)	C18B—C17B—C16B—C21B	0.0
C9—C6—C7—C1	173.5 (7)	C18B—C17B—C16B—C15	−178.6 (14)
C4—C1—C7—O3	−32.7 (9)	C16A—C15—C16B—C21B	−69 (11)
C2—C1—C7—O3	−148.0 (7)	O3—C15—C16B—C21B	90.9 (13)
C4—C1—C7—C8	−160.7 (7)	C16A—C15—C16B—C17B	109 (11)
C2—C1—C7—C8	84.0 (8)	O3—C15—C16B—C17B	−90.5 (10)
C4—C1—C7—C6	84.0 (8)	C2—C3—O1—C4	28.3 (10)
C2—C1—C7—C6	−31.2 (9)	C1—C4—O1—C3	−12.3 (10)
C5—C6—C9—C14	28.5 (13)	C2—C5—O2—C6	−101.8 (7)
O2—C6—C9—C14	98.1 (10)	C9—C6—O2—C5	−116.9 (8)
C7—C6—C9—C14	−119.2 (10)	C7—C6—O2—C5	97.1 (7)
C5—C6—C9—C10	−150.6 (8)	C16A—C15—O3—C7	176.7 (9)
O2—C6—C9—C10	−81.0 (9)	C16B—C15—O3—C7	174.6 (8)
C7—C6—C9—C10	61.7 (11)	C8—C7—O3—C15	56.0 (8)
C14—C9—C10—C11	0.9 (13)	C6—C7—O3—C15	174.7 (6)
C6—C9—C10—C11	−179.9 (8)	C1—C7—O3—C15	−71.1 (8)

### (III) (±)-(1aR,1bS,4aR,5S,5aR)-5-Benzyloxy-5-methyl-5a-phenylhexahydro-2H-oxireno[2',3':3,4]cyclopenta[1,2-c]furan Hydrogen-bond geometry (Å, º)

Cg6 is the centroid of the C16a–C21a ring.

**Table:**

*D*—H···*A*	*D*—H	H···*A*	*D*···*A*	*D*—H···*A*
C10—H10···O3	0.95	2.57	3.124 (10)	117
C8—H8*B*···O2^i^	0.98	2.58	3.462 (10)	150
C14—H14···O2^ii^	0.95	2.57	3.450 (11)	155
C4—H4*B*···*Cg*6^iii^	0.99	2.65	3.569 (10)	154

Symmetry codes: (i) *x*+1, *y*, *z*; (ii) *x*+1/2, −*y*+3/2, −*z*; (iii) −*x*+1, *y*+1/2, −*z*+1/2.
